# Functional and dysfunctional conformers of human neuroserpin characterized by optical spectroscopies and Molecular Dynamics

**DOI:** 10.1016/j.bbapap.2014.10.002

**Published:** 2015-02

**Authors:** Rosina Noto, Maria Grazia Santangelo, Matteo Levantino, Antonio Cupane, Maria Rosalia Mangione, Daniele Parisi, Stefano Ricagno, Martino Bolognesi, Mauro Manno, Vincenzo Martorana

**Affiliations:** a Institute of Biophysics, National Research Council of Italy, Palermo, Italy; b Department of Physics and Chemistry, University of Palermo, Palermo, Italy; c Department of Biosciences, Institute of Biophysics CNR, Italy and CIMAINA, University of Milano, Milan, Italy

**Keywords:** Neuroserpin, Serpin, Conformational disease, Fluorescence, Molecular Dynamics, Circular dichroism

## Abstract

Neuroserpin (NS) is a serine protease inhibitor (SERPIN) involved in different neurological pathologies, including the Familial Encephalopathy with Neuroserpin Inclusion Bodies (FENIB), related to the aberrant polymerization of NS mutants. Here we present an *in vitro* and *in silico* characterization of native neuroserpin and its dysfunctional conformation isoforms: the proteolytically cleaved conformer, the inactive latent conformer, and the polymeric species. Based on circular dichroism and fluorescence spectroscopy, we present an experimental validation of the latent model and highlight the main structural features of the different conformers. In particular, emission spectra of aromatic residues yield distinct conformational fingerprints, that provide a novel and simple spectroscopic tool for selecting serpin conformers *in vitro*. Based on the structural relationship between cleaved and latent serpins, we propose a structural model for latent NS, for which an experimental crystallographic structure is lacking. Molecular Dynamics simulations suggest that NS conformational stability and flexibility arise from a spatial distribution of intramolecular salt-bridges and hydrogen bonds.

## Highlights

• Different neuroserpin conformers are characterized *in silico* and *in vitro*.
• Emission spectra of aromatic residues yield a distinct conformational fingerprint.
• A novel spectroscopic tool for selecting serpin conformers *in vitro* is proposed.
• The structure of latent NS is computationally modeled and experimentally validated.
• The pattern of intramolecular bonds explains the stability of neuroserpin conformers.

## Graphical abstract

**Figure:**
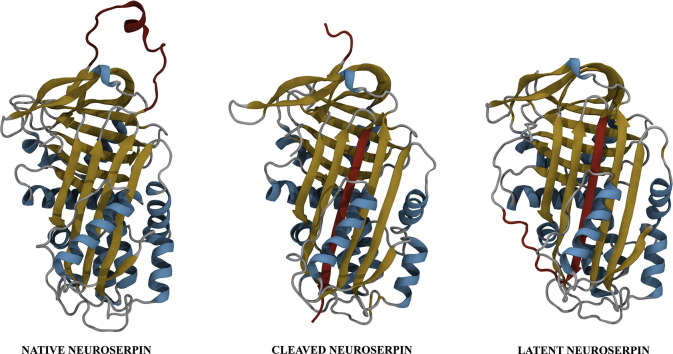

## 1 Introduction

Neuroserpin (NS) is an axonally secreted protein [1], belonging to the Serpin family (SERine Protease INhibitor) [2]. NS is an inhibitor of tissue-type plasminogen activator, with a role in physiological processes [3] such as synaptic plasticity, memory, or sterol metabolism [4], as well as in pathological contexts, such as Alzheimer disease [5]. Site mutations in NS amino acid sequence cause an autosomal dominant dementia, known as Familial Encephalopathy with Neuroserpin Inclusion Bodies (FENIB) [6], related to aberrant deposition of NS polymers [7–10]. Such pathology, characterized by an evident genotype–phenotype correlation [11], is a striking example of a class of conformational diseases, the serpinopathies, related to specific serpin site mutations, as in the case of the most common α_1_-antitrypsin deficiency [12].

Neuroserpin shares the typical serpin fold characterized by a large five stranded β-sheet (sA), partially covered by an α-helix (hF), and an exposed reactive central loop (RCL) that acts as a pseudo-substrate for the target protease [13]. Protease inhibition occurs when the RCL is cleaved at a specific site by the protease and is inserted as a new strand of the A β-sheet, while the protease remains covalently trapped as an acyl enzyme intermediate [14]. In the case of NS such a final complex is poorly stable: the protease is eventually released, while NS remains in a stable loop-inserted “cleaved” isoform [15]. Alternatively, as for other serpins, NS may insert the uncleaved RCL into the A β-sheet, thus achieving the “latent” isoform, a permanently inactive state that is more stable than the native one [16]. On the other hand, the so called “mouse-trap” mechanism, which is at the heart of the serpin inhibition mechanism, can lead to linear protein polymer production, through the serial intermolecular exchange of RCLs among neighboring serpin molecules [17]. The first polymerization mechanism proposed to explain polymerization considered the insertion of the RCL of one molecule into the activated A β-sheet of a nearby molecule [18,19]. Recently, other models have been proposed, involving extensive domain swapping and major unfolding of the polymer forming molecules [20,21].

In recent studies, we showed how the structural details of dysfunctional NS conformers, polymer and latent NS, depend upon thermodynamic and environmental conditions [22,23], in keeping with other studies [24]. Further, we proposed that the mechanism of NS polymerization is rate limited by the formation of an intermediate conformation prone to dimerization [25,26], as in other serpins [27], and is controlled by a peculiar link between depolymerization and concomitant latentization [25]. Further, Onda and coworkers have recently identified a refolding intermediate leading to the formation of polymers alike those formed by native NS [28].

Detailed knowledge on serpin structure and dynamic behavior (intermediate conformations, conversion to latency, polymerization) is fundamental to design molecules for the treatment of diseases dependent on serpin polymerization [29]. However, the structure of the pathological polymers is still a matter of debate [17], and the structural information concerning a dysfunctional state such as the latent state is still elusive, only a few crystal structures being available [30–34].

Beyond its relevance in human pathology, NS is an excellent model for biophysical studies of serpin polymerization due to its close structural homology with the archetypal serpin α1-antitrypsin (AAT) [15], but also in view of its capability of forming polymers and latent conformers under thermal stress [8–10,22–26] or under acidic conditions [24], even in the wild-type form. Indeed, NS is relatively less stable than AAT, as marked by the achievement of fragile NS oligomers [25], and by the lability of the tPA–NS complex [15]. The determination of the crystal structures of the native NS [15,35] and of its cleaved form [15], along with recent results obtained by NMR and computer simulation [36], have helped in recognizing some of the key dynamical and structural features of NS.

Here, we extend the previous studies to all NS functional and dysfunctional conformations, by performing *in vitro* and *in silico* characterizations of native, cleaved, latent and polymeric NSs.

In particular, far-UV circular dichroism and intrinsic fluorescence measurements highlight the existence of distinct conformational fingerprints for the different NS conformers, and at same time a close analogy between the latent and cleaved NSs, in keeping with our model for the latent NS. A key feature is given by the emission spectra of ionized tyrosine residues, which are less contributed in the latent and cleaved form.

Moreover, we investigate the structural and dynamical differences characterizing the native, cleaved and latent conformers of NS, through molecular modeling techniques. In the absence of experimental 3D structures, a reasonable model is proposed for the latent NS, based on the corresponding conformer of AAT [30] and on the cleaved NS [15]. Our long equilibrium Molecular Dynamics (MD) simulations point out a hindered mobility and an enhanced stability (approximately 10–20 kcal mol^− 1^ free-energy) of the latent and cleaved NS conformations relative to the native one, in part revealed by a salt-bridged network.

## 2 Materials and methods

### 2.1 Production of different neuroserpin conformers

Recombinant NS was expressed and purified according to a previously published protocol [15,28]. Protein concentration was measured by UV absorption at 280 nm (extinction coefficient: 0.803 cm^− 1^ mg^− 1^ ml^− 1^, molecular mass: 46,280 g mol^− 1^). Latent and polymer NSs were produced by incubating a 20 μM solution of native NS at 55 °C for 2 h; the polymers were subsequently separated by size exclusion chromatography (SEC; Appendix A, Supporting Fig. A1). Cleaved NS was obtained by incubating a 200 μM solution of native NS at 37 °C for 1 h with trypsin (Sigma), applying a 1:10 protease–NS concentration ratio. The proteolytic reaction was blocked by prompt addition of soybean trypsin inhibitor (Sigma) at the final concentration of 150 μM. Cleaved NS was further purified by SEC. The clear-cut identification of NS conformers and the lack of native protein in the latent samples were assessed by native polyacrylamide gel electrophoresis, which showed that the non-native conformations migrate faster than native NS due to their more compact shapes [37] (Appendix A, Supporting Fig. A2).

### 2.2 Circular dichroism

Circular dichroism (CD) spectra were recorded, using 0.01 cm quartz cuvettes, on a J-815 spectropolarimeter (Jasco, Tokyo, Japan) equipped with a Peltier-type temperature-control system. The spectra were acquired with the average of 9 scans (3 nm bandwidth, 8 s response, 10 nm min^− 1^ scan rate) and baseline-corrected by subtracting a buffer spectrum. The mean residue differential extinction coefficient Δ*ε*_res_, in M^− 1^ cm^− 1^ units, was calculated from the observed ellipticity *θ*, in degrees, by the expression Δ*ε*_res_ = *θ*(*N*_res_*dc*32.982)^− 1^ [38], where *d* is the path length in cm, *N*_res_ = 410 is the number of residues in the recombinant NS used, and *c* is the protein molar concentration: 20, 21, 12.4 and 20 μM, for native, cleaved, latent and polymer NSs, respectively.

### 2.3 Intrinsic fluorescence

Fluorescence spectra were acquired at different excitation wavelengths (*λ*_ex_) over a 260–520 nm emission wavelength (*λ*) range using a 3 mm quartz cuvette and a Jasco FP-6500 spectrofluorimeter at room temperature (response 2 s, 1 nm excitation bandwidth, 3 nm emission bandwidth, 100 nm min^− 1^ scan rate), and baseline-corrected by subtracting a buffer spectrum. All the spectra were corrected for the instrument response (evaluated with an independent measurement of the emission spectrum of tryptophan in water [39,40]), and subtracted of the Rayleigh elastic peak. Emission spectra have been normalized with respect to their integral and then scaled in order to fit the tail (above 450 nm) of the emission spectrum measured with excitation at 295 nm. The rationale of this treatment is that, upon excitation at 295 nm, only tryptophan residues are excited. At the same time, tyrosine emission above 450 nm is negligible. The resulting *Ī*(*λ*_ex_;*λ*) spectra are directly related to the spectra *S*_Trp_(*λ*) and *S*_Tyr_(*λ*) of Trp and Tyr residues, respectively: *Ī*(*λ*_ex_;*λ*) = *S*_Trp_(*λ*) + *S*_Tyr_(*λ*)*A*_Tyr_(*λ*_ex_), where *A*_Tyr_(*λ*_ex_) is the integral of Tyr spectra.

### 2.4 Molecular Dynamics simulations

The NS models corresponding to each state were solvated in rectangular simulation boxes of 9.3 × 7.3 × 7.2 nm^3^ for native NS, 6.8 × 7.9 × 8.6 nm^3^ for cleaved NS, and 6.7 × 7.7 × 8.7 nm^3^ for latent NS. Each system contained about 12,000–13,000 TIP3 water molecules [41], with 15 potassium ions to neutralize the protein net charge, adding up to a total of 42,000–45,000 atoms. The water box was created to secure a solvent layer of 1 nm in each direction. The tautomeric form of the His residues appropriate for neutral pH was chosen with the help of a VMD tool. After adequate minimization and equilibration steps (Appendix A, Supporting Table A1), MD trajectories were generated, using the NAMD2 package [42] and the Charm22 force field [41] in the NPT ensemble at 300°K and atmospheric pressure; periodic boundary conditions were employed, Van der Waals and Coulomb interactions were truncated using a switch function at a cutoff value of 1 nm, while long-range electrostatic interactions were evaluated by the Particle Mesh Ewald method. The SHAKE algorithm was used to constrain the bond lengths of heavy atoms, allowing the use of a 2 fs time step. A simulation of the same system in the NVE ensemble was performed to check for energy conservation, to ensure that the equations of motion were accurately solved.

### 2.5 Computational analytical tool

Different software packages and computational tools were used for the analysis of secondary structure elements, Solvent Accessible Surface Area, hydrogen bonds, and essential modes (Appendix A, Table A2).

## 3 Results and discussion

**Fig. 1:**
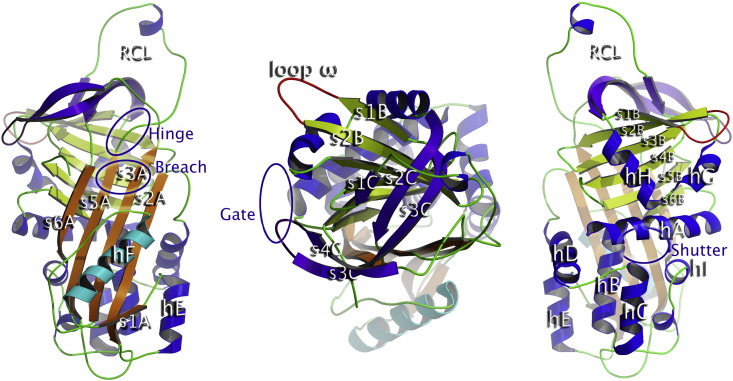
Three views of the human NS. The cartoon representation presents the nomenclature of secondary structure elements. Left: the classical presentation of serpins with F α-helix on the front. Center: 90° rotation across the horizontal axis; the RCL is on the front. Right: 180° rotation around the vertical axis, with the α-helix F on the back. The main structural features are highlighted in colors: A β-sheet (orange), B β-sheet (lime), C β-sheet (prune), RCL (green–blue), and F α-helix (cyan) and Ω-loop (red). Some key regions are circled.

The serpin tertiary structure typically hosts three beta-sheets, labeled A, B and C, and nine alpha-helices. Fig. 1 shows a cartoon representation of NS in its native conformation, where such main secondary structure features are highlighted. Three regions are known to be important for the serpin conformational changes and are located at the top, center-top, and at the center of the A β-sheet respectively [13]. Such key regions are conventionally identified as: (i) the hinge, considered a switch point for RCL insertion; (ii) the breach, the onset point for RCL insertion; and (iii) the shutter, behind the center of the A β-sheet containing also part of the s6B strand and the top of helix B. We monitored a fourth important region: (iv) the gate region, located between the turn linking the G helix with the s3B strand (gate-turn1) and the turn linking strands s3C and s4C (gate-turn2). Insertion of the RCL into the A β-sheet without any cleavage (inactivation or latentization) requires it to pass through the gate region.

### 3.1 Characterization of NS secondary structure and validation of the latent model

The secondary structure contents of each of the three monomeric conformations (native, cleaved and latent), along with those of polymeric NS, were addressed by far-UV CD measurements. The CD spectra of both cleaved and latent NSs show a less intense negative band around 220 nm and 209 nm, relative to the spectrum of native NS, suggesting an increase in β-sheet structure [38]. Conversely, the CD spectrum of polymeric NS exhibits a band at lower wavelengths, typical of more disordered structures.

**Fig. 2:**
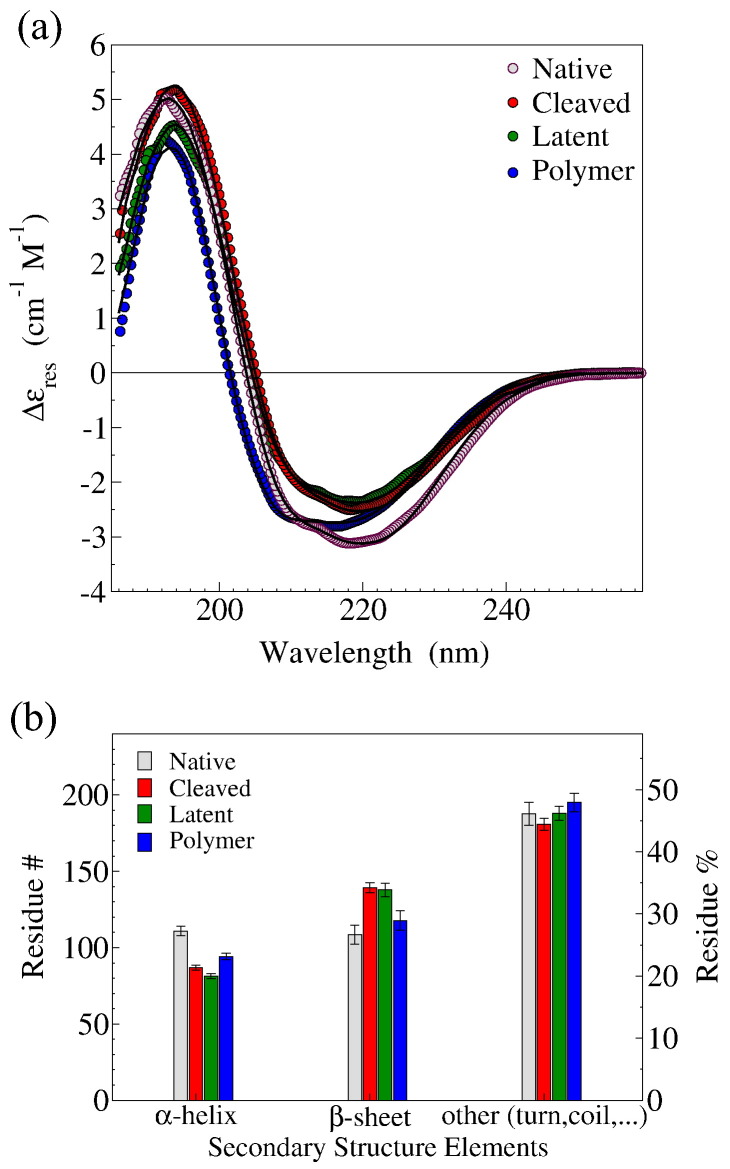
CD spectra of NS conformers. (a) Far UV CD spectra of native (gray circles), cleaved (red circles), latent (green circles) and polymeric (blue circles) NSs; continuous black curves are the best fitting to the data. (b) Secondary structure contents reported as number of residues (#) or as overall residue percentage (%), resulting from CD spectra fitting, for native (gray bars), cleaved (red bars), latent (green bars) and polymer (blue bars) NSs.

A more quantitative estimate was obtained by fitting the CD spectra using the CDPro software package [43] (Fig. 2a), confirming a higher extent of β-structure in the latent and the cleaved forms (Fig. 2b). Interestingly, the difference between the polymer and native NS structure is in keeping with results previously reported by us and others [25,27,35], and consistent with the generally accepted idea that serpin polymers should not involve extensively unfolded molecules [17,29].

### 3.2 Characterization of NS overall structure and identification of the latent conformation

We structurally characterized the different NS conformations by measuring the intrinsic fluorescence emission, which in proteins is dominated by tryptophan (Trp) and tyrosine (Tyr) residues [39]. Wild-type NS hosts three Trp and fourteen Tyr residues. In order to separate their spectral contributions, the emission spectra were measured at two different excitation wavelengths: 295 nm, where only Trp contributes, and 275 nm, close to the absorption maximum of both Trp and Tyr.

**Fig. 3:**
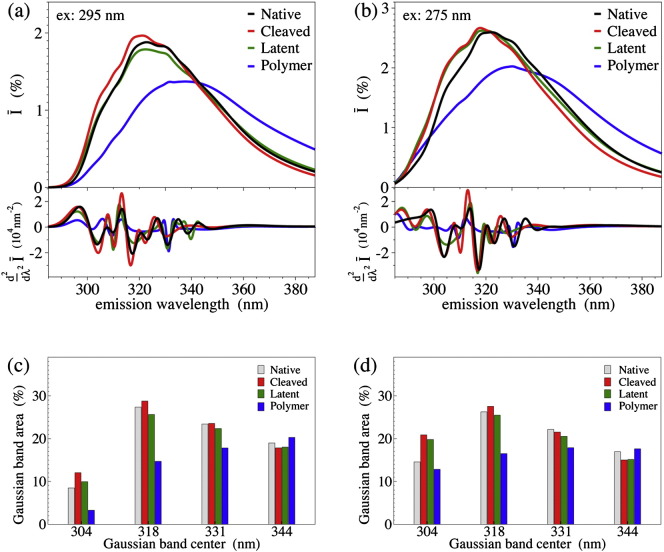
Fluorescence spectra of NS conformers. Panels (a) and (b): emission spectra of native (black), cleaved (red), latent (green) and polymeric (blue) NSs, with excitations at 295 nm (a) and 275 nm (b). At the bottom of panels (a) and (b): second derivative of the emission spectra. Panels (c) and (d): fractions of the different Gaussian components (colors as in the legend).

The intrinsic fluorescence emission spectra of the different NS conformers, *Ī*(*λ*_ex_;*λ*), are displayed in Fig. 3a and b for the excitation at 295 and 275 nm, respectively. Emission spectra were also measured at different excitation wavelengths around the absorption peak (270 and 280 nm), recording analogous behaviors, since the relative absorption of Tyr and Trp is alike around the maximum (data not shown). By observing the second derivative minima (at the bottom of Fig. 3a,b) we note that the emission spectra are highly structured. We may single out five main contributions, centered at 304, 318, 331, 344 and 360 nm, respectively (other minor bands could be considered at 310, 326 and 352 nm). Note that the band at 360 nm is included to take into account the non-symmetric shape of the emission band and to correct the intrinsic bias introduced by an analysis in terms of Gaussian components. These components match the scheme of discrete classes of Trp residues predicted to be most probable in proteins [39,44], which include: (i) Class A, *λ*_m_ = 308 nm, buried Trp; (ii) Class S, *λ*_m_ = 316 nm, buried Trp forming exciplexes (H-bonded complexes in the excited state); (iii) Class I, *λ*_m_ = 331 nm, buried Trp forming multiple exciplexes; (iv) Class II, *λ*_m_ = 341 nm, partially exposed Trp; and (v) Class III, *λ*_m_ = 351 nm, extensively exposed Trp. The analysis in terms of Gaussian components (Appendix A, Supporting Fig. A3) allows to quantify the contribution from each emission bands (Fig. 3c and d for the excitation at 275 and 295 nm, respectively).

The emission spectrum of polymeric NS exhibits a clear red-shift relative to the native NS, along with a conspicuous intensity quenching (although the latter effect is not evidenced in the normalized spectra shown in Fig. 3a,b). This indicates that the Trp residues of polymeric NS are more exposed to the solvent than those in the monomeric conformers, in agreement with previous studies by us and others [25,28]. At the opposite, the emission spectrum of cleaved NS is slightly blue-shifted, due to the more pronounced band at 304 nm. This is evident from inspection of the 295 nm excitation *Ī*(*λ*_ex_ = 295 nm; *λ*), and may be ascribed to a more constrained Trp environment, likely due to a damping of conformational fluctuations, as evidenced by MD simulations. The band at 304 nm may be due to Tyr emission, as evidenced by the spectra with 275 nm excitation *Ī*(*λ*_ex_ = 275 nm; *λ*) (Fig. 3b,d). This characteristic shoulder at 304 nm caused by both Trp and Tyr emission stands as a characteristic feature of cleaved NS, which allows distinguishing the cleaved conformers from the native one.

The latent emission spectrum with excitation at 295 nm is largely not distinguishable from the native one. In particular, the low wavelength bands of native and latent NSs are comparable, suggesting negligible perturbation of the Trp environments in terms of exposure or reduced mobility. Indeed, no evident spectral differences have been observed so far in the emission of different NS or serpin conformers, with the exception of the trivial red-shift in polymers or partially folded intermediates [35,45–47]. The picture changes at the 275 nm excitation. Here, the latent emission spectrum is closely similar to that of the cleaved one (Fig. 3b,d). Therefore, Tyr emission allows identifying the latent conformation and marks a spectroscopic fingerprint for this conformer. Latent and polymer NSs were also formed by incubation at higher temperature exhibiting the same spectroscopic features of those formed at 55 °C.

The ratios in the Gaussian band area at 304 and 331 nm for the native, cleaved and latent NSs are 0.36, 0.51, and 44, respectively, at 295 nm, while they increase to 0.66, 0.97, and 0.96 at 275 nm. In other words, in the latent and cleaved NSs the two bands become equivalent. An analysis-free parameter, unbiased by both instrumental calibration and data normalization is the ratio *R* of the emission intensities at 304 nm (with excitations at 275 and 295 nm) relative to the correspondent intensities at 331 nm: namely *R* = *I* (275 nm; 304 nm) / *I*(275 nm; 331 nm) ∙ [*I*(295 nm; 304 nm) / *I*(295 nm; 331 nm)]^− 1^ (see Table 1).

The *R*-values for the native, cleaved and latent NSs are *R* = 1.33, 1.42, and 1.55 respectively. This parameter, along with the overall shape of the emission spectrum excited at 275 nm, may represent a useful tool for serpin studies, since it allows a possible non-invasive identification of serpin monomeric conformers, besides the classical native polyacrylamide gel electrophoresis approach [37].

### 3.3 Tyrosine emission spectra and interaction network

**Fig. 4:**
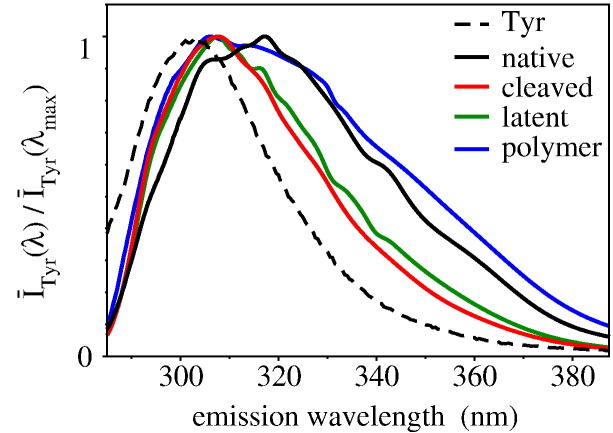
Tyr emission spectra. Solid curves are native (black), cleaved (red), latent (green) and polymer (blue) NSs. Dotted line is a standard emission spectrum of Tyr in water. Spectra are normalized to the maximum.

The shape of the emission spectra of the NS conformers poses the question of the origin of the differences in Tyr emission. Since Tyr emission is relatively insensitive to the local environment [39], it could be excluded that the observed differences arise from a different solvent exposure of Tyr residues. Also, although a Tyr–Trp resonant energy transfer should display a high efficiency in NS, this is not the origin of the spectral differences, since the Tyr spectra integrals have comparable values [48] (Appendix A, Supporting Fig. A4). Fig. 4 shows the Tyr spectra obtained by subtracting the spectra of Fig. 3a from the spectra of Fig. 3b: *Ī*_Tyr_(*λ*) = *Ī*(*λ*_ex_ = 275 nm; *λ*) − *Ī*(*λ*_ex_ = 295 nm; *λ*). The spectral shapes are wider than the typical Tyr spectrum in aqueous solvent, and such a broadening is enhanced in native and polymeric forms and reduced in cleaved and latent conformations. One may represent these spectra with a proper Tyr band centered at 304 nm and a second band around 340 nm, which is typical of the emission of tyrosinate, which may depend on a decrease of the residue pKa in the excited state [39]. The proximity of a positive charge to the Tyr hydroxyl group may also help lowering the intrinsic Tyr pKa promoting the formation of tyrosinate. The only basic residue having an average distance from a Tyr within 3 Å is Arg362 on the RCL, which is close to Tyr218 on the s3C strand in native NS, and displaced upon inactivation in the cleaved and latent NSs. Other differences in the location of Tyr residues between native and cleaved NSs may be found in residues 291 (on strand 2C) and 367 (in the RCL), which become more solvent exposed upon RCL displacement, as well as on Tyr185 on the strand s3A, which is close (below 3 Å) to Glu343 on the strand s5A in the native NS, while such a distance increases to 5 Å in the other two monomeric conformers. However, the action of the negatively-charged carboxyl group of Glu343 as a competitor for the Tyr hydrogen is not the most likely, given the intrinsic low pK of glutamic acid. The tyrosinate band is also relevant in the polymeric NS, however it is not possible to speculate about the polymer model in the absence of structural details.

### 3.4 MD simulations: starting coordinates and latent modeling

All-atom MD simulations were performed on the three NS states solvated with water molecules. The initial molecular structures for MD simulations were based on the X-ray crystallographic coordinates of NS by Ricagno et al. [15] for the native (PDB ID: 3F5N) and cleaved NSs (PDB ID: 3F02), and on analogy with the AAT structures [49,50] (for details see Appendix A, Supporting Table A1). Since no crystal structure is available for the NS latent form, we modeled the latent NS in a cleaved-like conformation, that is according to the cleaved NS crystal structure [15], with the full insertion of the RCL into the A β-sheet as strand s4A (Appendix A, Supporting Fig. A5). The dangling, presumably disordered segment, composed by the s1C strand and part of the RCL, was modeled according to a crystallized latent form of AAT (PDB ID: 1IZ2) [30]. A 3D molecular model for the latent NS is reported in Appendix A along with 3D models for native and cleaved NSs used in MD simulations.

### 3.5 MD simulations: NS structure, stability and salt bridges

The three NS conformers displayed a remarkable conformational stability throughout the 45 ns long trajectories, which remained close to the crystallographic structures, with no sign of unfolding, and with Root Mean Square Displacement (RMSD) values in the order of 2.3 Å.

(Appendix A, Supporting Fig. A6). Also the calculated radii of gyration remained closely comparable with those calculated from the crystal structures and did not show specific trends (Appendix A, Supporting Table A3).

The secondary structure fractions calculated from CD spectra (CDPro) were compared with those assigned from the crystallographic coordinates, or from the MD simulations by means of the STRIDE algorithm [51] (Fig. 2b, Table 1). Within an overall good agreement, it is worth noting that the simulation results lay in between the results obtained from the CD spectra and the crystallographic data, in keeping with the concept that MD simulations help in relaxing part of the constraints posed by packing in the crystal lattice. A slight overestimate of the α-helix content of cleaved and latent NSs both in the *in silico* and *in crystal* analyses is likely due to a correlated underestimate of disordered structures, which are not resolved in the crystal structure. The slightly lower content in the β-sheet structure (about 10 residues) of the latent form with respect to the cleaved one, although being at the limit of uncertainty, may be ascribed to partial unfolding of strand s1C and to the loss of structure at the ends of strands s5A and s4A.

**Fig. 5:**
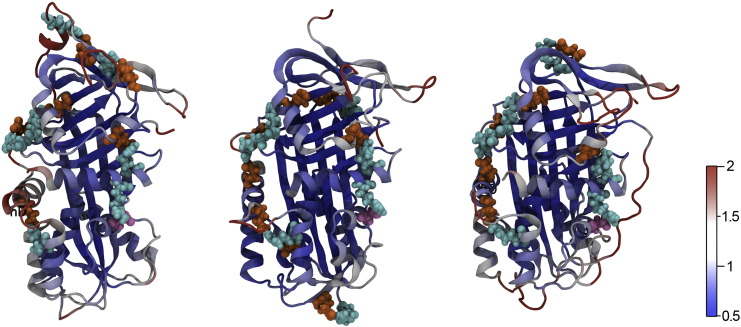
RMSF of the NS conformers. C_α_ RMSF mapped onto the average NS structure colored according to displacement during 45 ns, for the three NS conformers (with helix F on the back as in the right panel of Fig. 1): native (left), cleaved (center), latent (right). The structural elements are color-coded from blue (RMSF: 0.5 Å, stable) to red (RMSF: 2.0 Å, flexible), as in the color scale bar on the right. Note the hD helix flexibility, which is higher in the native than in cleaved and latent forms. The most stable salt bridges are represented as colored spheres: arginine (orange), glutamate (cyan), and aspartate (pink).

Time-averaged residue-based Root Mean Square Fluctuations (RMSF), calculated after alignment of the MD generated structures with the average structure, are color-mapped onto the protein structures in Fig. 5. Apart from turns, termini and loops, which are trivially flexible in all conformers, native NS shows a slightly higher conformational flexibility, as expected in view of the larger number of non-native contacts (Appendix A, Supporting Table A3). Such a flexibility is notably related to the D and E helices, in keeping with the fact that these regions are disordered in the crystal structures [15,28].

A rationale for such different flexibility can be found in the stable salt bridges, represented as colored spheres in Fig. 5, to highlight their spatial pattern. Indeed, the cleaved and latent conformers show a continuous chain of salt bridges involving the D helix, which anchor different regions and may damp fluctuations (Fig. 5). An additional noticeable cluster of salt bridges stabilizes the A, I and G helices and is present in all the three NS conformers, being more extended in cleaved NS together with two complex salt bridges, which are known to play key roles in the protein stability [52] (Appendix A, Supporting Table A4).

A partial opening of the breach region at the top of s3A and s5A strands occurs around *t* = 42 ns in the native NS conformer [36], while no similar events were detected in the trajectories of the cleaved and latent NSs. The NS breach region is pivotal for the mechanism of RCL insertion, and indeed it is a highly conserved structural feature across the serpin family [53]. Such observation prompted us to extend the relative MD simulation up to about 100 ns, to better sample the associated conformational rearrangements. This event is accompanied by the formation and disruption of hydrogen bonds and salt bridges both in the breach region and in other regions of the protein (Appendix A, Supporting Fig. A7). Moreover, Essential Mode Analysis [54] confirms that such opening is related to the main protein collective mode; in particular it highlights a suggestive correlation between opening of the breach region and large collective movements of the NS loops (Appendix A, Supporting Fig. A8).

### 3.6 MD simulations: free-energy changes related to neuroserpin inactivation

Solvent exposure of the protein surface for the different NS conformers was measured by calculating the Solvent Accessible Surface Area (SASA) and distinguishing the contribution due to polar atoms (hydrophilic contributions) and non-polar atoms (hydrophobic contributions). Surprisingly, the differences among the three conformers are small, notwithstanding the important associated conformational transitions. Indeed, the maximum difference is in the order of 500 Å^2^, *i.e.* corresponding to the exposure of a few residues. Cleaved NS has both the lowest non-polar SASA and a very high polar contribution. Latent NS shows the largest total area value mainly due to the excess SASA of non-polar atoms, which is reduced in cleaved NS. In general, we find that some kind of compensating effect levels off the differences that could be naively expected. For example, the latent conformer compensates the decrease in SASA, due to RCL insertion, with a larger surface exposed by residues in the B and C β-sheets. In particular, unfolding of the s1C strand contributes strongly to its non-polar excess surface area. We may estimate the free-energy differences due to apolar and polar surfaces by taking the native NS structure as a reference state, and referring to the empirical expression [55]: Δ*G*_SASA_ = *σ*_apol_ΔSASA_apol_ − *σ*_pol_ΔSASA_pol_, where *σ*_apol_ = 49.6 cal mol^− 1^ Å^− 2^ and *σ*_pol_ = 19.1 cal mol^− 1^ Å^− 2^. Due to their more favorable SASA, the cleaved and latent NSs are more stable than the native NS, with the following free energy contributions Δ*G*_SASA_(cleaved) = − 14.4 kcal mol^− 1^ and Δ*G*_SASA_(latent) = − 6.5 kcal mol^− 1^.

**Fig. 6:**
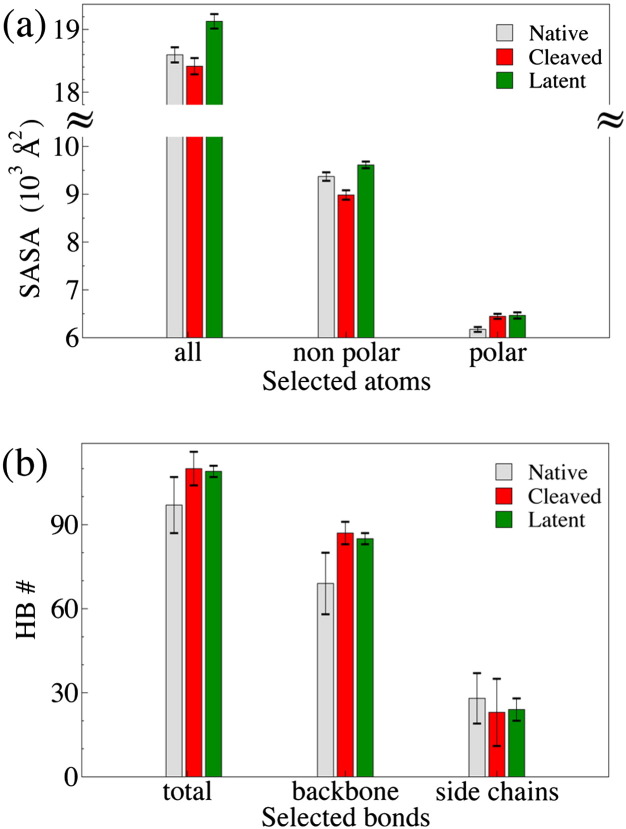
Comparison of structural features for the three NS forms, calculated from the MD simulated models. (a) Solvent Accessible Surface Area (SASA) calculated for all, nonpolar and polar atoms. (b) Number of hydrogen bonds (HB) with the > 50% occupancy for total, backbone and side-chain HB.

Hydrogen bonds (HBs) play an important role in protein structural stability and functionality [56,57]. Fig. 6 shows the HBs involving backbone or side-chain atoms for the three NS conformers. The differences are small but significant, in particular for the backbone HB, where the cleaved and latent forms show an increased number of HBs, mainly due to the interaction of the strands s5A and s3A with the new s4A strand. The latter strands form anti-parallel beta strands, which typically host stronger hydrogen bonds than those in parallel beta strands, as s3A and s5A are in native NS. An opposite trend is observed for side-chain HBs, but with differences that fall within the error bars. If one assigns a value of 0.5 kcal mol^− 1^ for the stabilizing free energy of each HB [58], the free-energy differences due to HBs with respect to native NS are: Δ*G*_HB_(cleaved) = − 5.5 kcal mol^− 1^ and Δ*G*_HB_(latent) = − 4.0 kcal mol^− 1^, suggesting that the cleaved and latent NSs are partly stabilized by intra-molecular H-bonding

As a further consideration, it is conceivable that some residue side-chains are restrained when NS assumes a less flexible conformation, thus decreasing the configurational entropy. We calculated such an entropic contribution following the approach of Pickett et al. [59], which consists in estimating the change in relative surface exposure and in weighting these changes with empirical coefficients on a residue-type basis [58]. The estimated terms for cleaved and latent NSs amount to Δ*G*_S_(cleaved) = 1.8 kcal mol^− 1^ and Δ*G*_S_(latent) = 1.3 kcal mol^− 1^, respectively. Thus, by bringing together the three free energy contribution described above, we estimate a total free energy gain of Δ*G*(cleaved) = − 18.1 kcal mol^− 1^ for the conversion of native NS into the cleaved conformer, and Δ*G*(latent) = − 9.2 kcal mol^− 1^, for the native to latent conformer transition. Although these estimated values are based on a series of empirical assumptions, they appear to capture the overall stability ranking of the three NS conformers, as determined by HD exchange MS, NMR or optical spectroscopy in thermal and chemical denaturation experiments [13,17,22,60,61].

## 4 Conclusions

Native NS, as other serpins, is natively folded in a metastable state that may convert into other conformational isoforms [16], namely the cleaved NS, resulting from protease cleavage during NS inhibitory activity, and the latent NS, a dysfunctional conformation inactive for inhibition, typically obtained by RCL insertion into the main NS A β-sheet without cleavage. The formation of pathological polymers is also intrinsically related to serpin metastability, although the actual intermolecular assembly model of the NS moieties is still debated [17]. In this scenario, we used Molecular Dynamics and optical spectroscopies to characterize *in silico* and *in vitro* the different NS conformers and to assess their structural and dynamical properties, as well as their free-energies.

Characteristic differences were observed in the emission spectra of the various NS conformers, related to the interaction patterns of tyrosine residues. Indeed, when only the NS Trp residues are excited at 295 nm, minor differences, if any, are observed in the emission spectra of the different monomeric conformers. On the contrary, by exciting at 275 nm, where tyrosine residues are also excited, emission spectra yield a clear fingerprint of the different conformers, and in particular allow to distinguish latent from native NS. This observation is particularly relevant as it offers a simple spectroscopic tool for the selection of NS conformers, which is currently performed *a posteriori* by gel electrophoresis. In view of its simplicity and generality, the possibility of transferring such spectroscopic approach to other serpin molecules appears attractive.

In the absence of a crystal structure for latent NS, MD simulations and computational modeling enabled us to propose a model for the structure of latent NS, based on its structural homology between cleaved NS [15] and latent AAT [30]. The model was validated by optical spectroscopy: indeed, the overall good agreement between secondary and tertiary structure elements obtained from the *in vitro* and *in silico* analyses, along with the conserved secondary structures observed in latent and cleaved NSs, can be considered as a validation of the proposed latent conformation which was modeled on the basis of the cleaved NS structure.

The NS structures obtained with Molecular Dynamics simulations proved very stable, with no significant deviations from the available crystallographic models [15,28], in keeping with the previous MD study of native NS [36]. Cleaved and latent NSs are more stable than native NS, with a free energy gain of − 18.1 kcal mol^− 1^ and − 9.2 kcal mol^− 1^, respectively, as expected for secondary structure changes in proteins or peptides [62]. Our calculations indicate that such stabilization arises from the hydrogen bond network formed upon RCL insertion, and from differences in polar and apolar Solvent Accessible Surface Areas and configurational entropy. The Root Mean Square Fluctuations analysis evidenced, for latent and cleaved NS, an increased rigidity in a region involving the D helix (Fig. 6), due to the formation of a network of stable salt bridges.

In summary, the present study proposes a simple non-invasive spectroscopic method to diagnostically distinguish between NS conformations. The transferability of this tool to other serpins may be considerably useful for biophysical and biochemical studies. The MD simulation further confirms the small structural differences among monomeric NS conformations and crucially provide a rationale for their different dynamics and thermodynamics stability.

## Figures and Tables

**Table 1:** Secondary structure analysis. Residues are assigned to the main secondary structure elements of the different NS conformers based on analysis of the crystallographic coordinates, MD simulations and CD data.

	*α*-Helix	*β*-Sheet	Other	
Native	111 ± 3	108 ± 6	188 ± 7	*in vitro*^1^
	107 ± 7	115 ± 7	185 ± 14	*in silico*^2^
	100	118	189 ⁎	*in crystal*^3^
	116	119	172 ⁎	*in crystal*^4^
Cleaved	087 ± 2	139 ± 3	181 ± 4	*in vitro*^1^
	106 ± 7	135 ± 7	166 ± 14	*in silico*^2^
	108	145	154 ⁎	*in crystal*^5^
Latent	081 ± 2	138 ± 4	188 ± 5	*in vitro*^1^
	102 ± 6	125 ± 6	180 ± 12	*in silico*^2^
Polymer	094 ± 2	118 ± 4	195 ± 6	*in vitro*^1^

⁎ Including not resolved residues.

1 Calculated by CDPro from CD spectra.

2 Calculated by STRIDE from MD simulation.

3 Calculated by STRIDE from PDB ID: 3F5N[15].

4 Calculated by STRIDE from PDB ID: 3FGQ[27].

5 Calculated by STRIDE from PDB ID: 3F02[15].

## Appendix A Supplementary data

Supplementary material 1 Supporting experimental and simulation data (8 figures and 4 tables).

Supplementary material 2 Latent Neuroserpin PDB.
