# Supplementary material for: Functional and dysfunctional conformers of human neuroserpin characterized by optical spectroscopies and Molecular Dynamics
Source: Biochim Biophys Acta. 2015 Feb;1854(2):110–7. doi: 10.1016/j.bbapap.2014.10.002 (PMC4332418; doi:10.1016/j.bbapap.2014.10.002)
Supplement: Supplementary material 1 — Supporting experimental and simulation data (8 figures and 4 tables). [file mmc1.pdf]

Functional and dysfunctional conformers of human neuroserpin characterized by optical spectroscopies and Molecular Dynamics

- Supporting Information - APPENDIX A -

Rosina Noto,[1†] Maria Grazia Santangelo,[1†] Matteo Levantino,[2] Antonio Cupane,[2] Maria Rosalia Mangione,[1] Daniele Parisi,[1,3,‡] Stefano Ricagno,[3] Martino Bolognesi,[3] Mauro Manno,[1,*] Vincenzo Martorana [1]

[1] Institute of Biophysics, National Research Council of Italy, Palermo, Italy;
[2] Department of Physics and Chemistry, University of Palermo, Palermo, Italy;
[3] Department of Biosciences, Institute of Biophysics CNR and CIMAINA, University of Milano, Milan, Italy.

[†] These authors equally contributed to the work.
[‡] Present address: Institute of Integrative Biology, University of Liverpool Liverpool, UK.
[*] Corresponding author: Mauro Manno, e-mail: mauro.manno@cnr.it
Institute of Biophysics, National Research Council of Italy, via Ugo La Malfa 153, 90146 Palermo, Italy. Phone: +39 091 6809305 Fax: +39 091 6809349

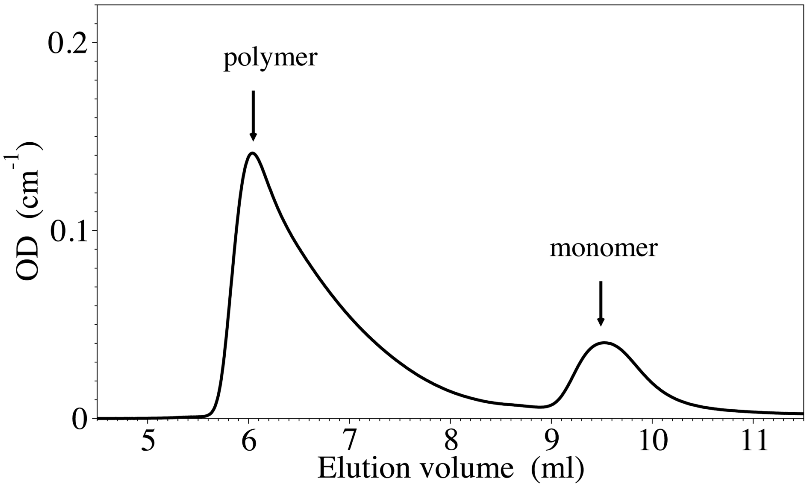

Supporting Figure A1. Chromatogram showing the separation of polymer and latent NS.

A 20 uM solution of native NS was incubated at 55 °C for 2 hours. At this temperature and protein concentration all the neuroserpin molecules are converted into non-native conformations, either latent or polymer [Santangelo et al. Proteins 80 (2012) 8-13; Noto, Santangelo et al. PLoS ONE 7 (2012) e32444]. The two species were separated by size exclusion chromatography (SEC) using either a BioSep-SEC-S3000 (300 x 7.80 mm) column (Phenomenex, Torrance, CA, USA), or a HiLoad 16/600 Superdex 200 column (GE Healthcare, Little Chalfont, UK), connected to a HPLC device, LC-2010 AT Prominence (Shimadzu, Kyoto, Japan), equipped with a UV-vis photodiode array detector. Similarly, latent and polymer NS were formed by incubation at other temperatures for different incubation times (namely: at 45 °C for 12 hours, 55 °C for 2 hours, 65 °C for 0.5 hours, 75 for 5 minutes, 85 for 2 minutes).

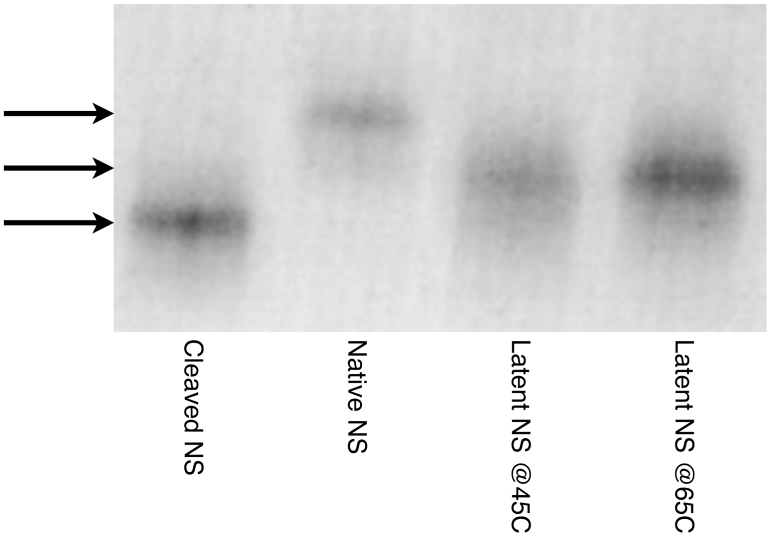

Supporting Figure A2. Native PAGE of native, cleaved and latent NS.

Monomeric samples were loaded onto a native polyacrylamide gel slabs consisting of a 7.5% acrylamide resolving gel and a 5% stacking gel. The native gel was run at 100V in standard sample and running buffers. The gel was then stained with Page Blue Protein Staining solution. The monomeric conformers, even if retaining the same molecular weight migrate differently on a native gel, since their hydrodynamic radius is slightly different. Indeed, latent and cleaved molecules adopt a more compact conformation resulting in a higher diffusion coefficient.

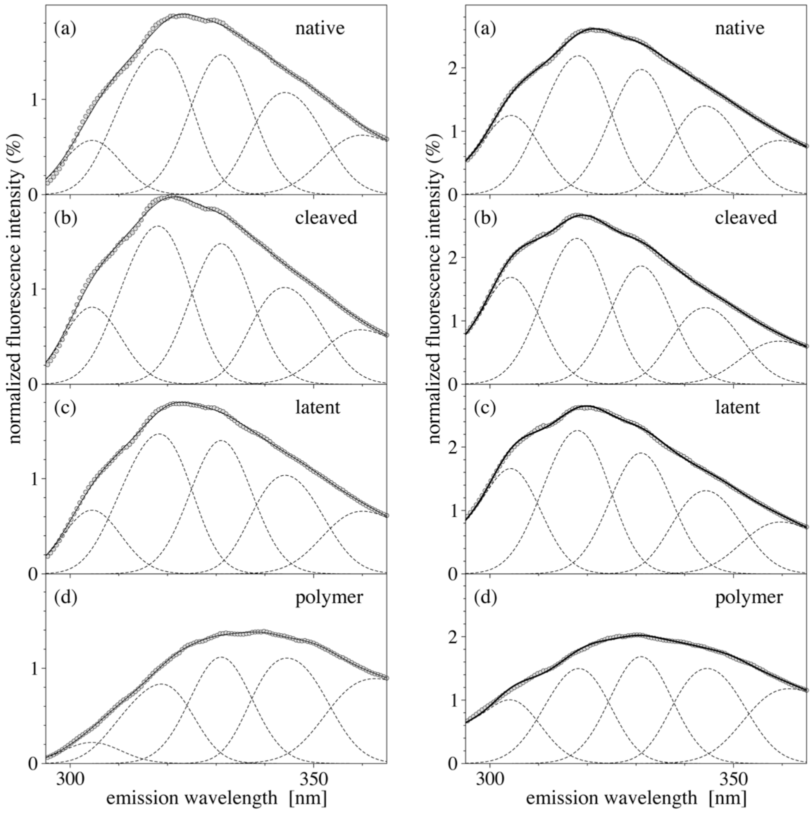

Supporting Figure A3. Gaussian analysis of fluorescence spectra.

Emission spectra are reported with excitation at 295 nm (left) and 275 nm (right), for (a) native, (b) cleaved, (c) latent, and (d) polymer NS. The circles are the experimental data, continuous lines are the fit , dashed lines are the different components. The measured intensity depends upon Trp and Tyr residues through their optical densities at the excitation wavelength, $OD_{Trp}(\lambda_{ex})$ *and* $OD_{Tyr}(\lambda_{ex})$, their quantum yields, Q_W and Q_Y, their normalized emission spectra, $S_{Trp}(\lambda)$ *and* $S_{Tyr}(\lambda)$, and the efficiency of the resonant energy transfer from Tyr to Trp, E_{ret}:

$$I(\lambda_{ex};\lambda) \sim S_{Trp}(\lambda)\, Q_{Tyr}[OD_{Trp}(\lambda_{ex})+E_{ret}\, OD_{Trp}(\lambda_{ex})] + S_{Tyr}(\lambda)\, Q_{Tyr}\, OD_{Tyr}(\lambda_{ex})\, [1-E_{ret}].$$

The scaled fluorescence intensities $\bar{I}(\lambda_{ex};\lambda)$ were obtained by normalizing for the integral of the emission spectrum and rescaling to fit the tail of the spectrum with excitation at 295 nm around 450 nm:

$$\bar{I}(\lambda_{ex};\lambda) = I(\lambda_{ex};\lambda)/I(\lambda_{ex};450nm) \cdot I(295nm;450nm)/\int I(295nm;\lambda)d\lambda.$$

Therefore, it is directly related to the the spectra of aromatic residues:

$$\bar{I}(\lambda_{ex};\lambda)=S_{Trp}(\lambda)+S_{Tyr}(\lambda)\, A_{Tyr}(\lambda_{ex})$$

$$A_{Tyr}(\lambda_{ex})=Q_{Tyr}/Q_{Trp}[1-E_{ret}][OD_{Trp}(\lambda_{ex})/OD_{Tyr}(\lambda_{ex})+E_{ret}]^{-1}$$

where $A_{Tyr}(\lambda_{ex})$ is a function of the ratio of Trp and Tyr optical densities at the excitation wavelength, which is close to 1 in the case of NS around the absorption maximum [$OD_{Trp}(275nm)/OD_{Tyr}(275nm)=0.92$].

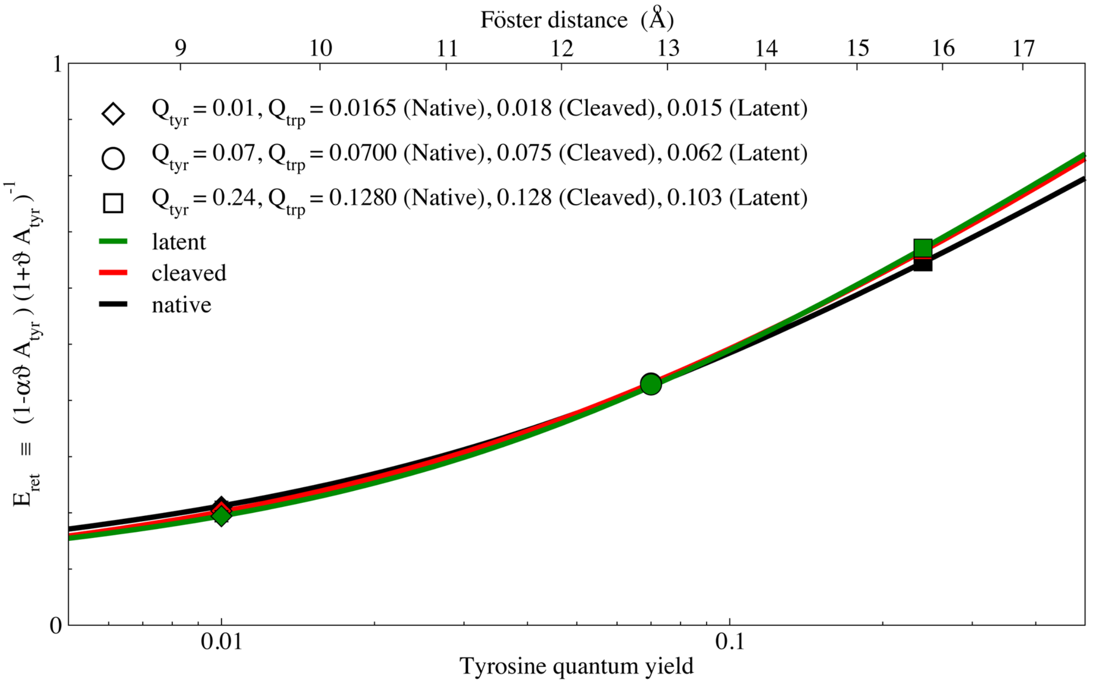

Supporting Figure A4. Resonant energy transfer analysis.

Tyrosine-Tryptophan resonance energy transfer efficiency E_{ret} is shown as a function of Föster distance R_F or tyrosine quantum yield Q_{Tyr} for native (black), cleaved (red) and latent (green) NS. Continuous lines are the average E_{ret} calculated form MD simulations assuming that all the tyrosines keep the same quantum yield: $E_{ret} = 1/14 \sum_{ij} [\ 1 + (R_{ij}/R_F)^6\ \kappa^2/\kappa_{ij}^2\ ]^{-1}$, where the sum is extended to all the couple of 14 Tyr and 3 Trp with distance R_{ij} and, orientation factor k_{ij} (the average orientation factor is taken as $\kappa^2=\langle\kappa_{ij}^2\rangle=2/3$). Here, we take $R_F{}^6 = Q_{Tyr}\, R_0{}^6$ and $R_0 = 20Å$, by assuming a protein refraction index of *1.45* and an overlap integral of Tyr and Trp of $4.8 \times 10^{-16}\ mol^{-1}\ cm^6$ [65]. E_{fret} has a monotonous dependence upon Q_{Tyr}, and is almost the same for each conformer.

The area of Tyr emission spectra in Fig. 6, $A_{Tyr}=\int\ \bar{I}(Tyr;\lambda)d\lambda$, could be used in principle to calculate the energy transfer efficiency. The diamond, squares and circles are calculated from experimental data of Tyr emission band area A_{Tyr} for given values of Tyr and Trp quantum yield, as specified in the legend:

$$E_{ret} = (\ 1 - OD_{Trp}/OD_{Tyr}\ Q_{Tyr}/Q_{Trp}\ A_{Tyr}\ )\ (\ 1 + Q_{Tyr}/Q_{Trp}\ A_{Tyr}\ )^{-1},$$

where OD_{Trp}/OD_{Tyr} is the ratio of Trp and Tyr optical density at the excitation wavelength 275 nm, that is close to 1 in the case of NS ($OD_{Trp}/OD_{Tyr} = 0.92$). Typical values yield energy transfer of about 40%, while lower (higher) values of Q_{Tyr} will point to lower (higher) E_{ret} values.

Supporting Table A1. Molecular Dynamics simulation details.

- MD calculations were performed on the IBM BCX/5120 cluster at CINECA.

- *Starting coordinates.* The initial molecular structures for MD simulations were based on the x-ray crystallographic coordinates by Ricagno et al. [15] (PDB: 3F5N and PDB: 3F02, for the native and cleaved NS, respectively). Starting from the monomer B of the crystallographic cell (the only one including the entire RCL coordinates), we reconstructed two missing short segments and a few residues in the native (res. 77-85, res 237) and cleaved forms (res. 82-84, res 95-101, res 234-235) by analogy with AAT (PDB: 1QLP [37] and PDB: 1D5S [38], for the native and cleaved AAT, respectively). The seven residues at the C-terminus of the crystallographic native structure were omitted for a direct comparison with the cleaved structure, where the segment has not been solved. The latent form was prepared according to the cleaved structure [15], which has a part of the RCL inserted into β-sheet A as strand s4A (Supporting Fig. A1). The dangling, presumably disordered segment, composed by the s1C strand and part of the RCL, was modeled by analogy with a crystallized latent form of AAT (PDB: 1IZ2 [30]).

- *Minimization, equilibration, production.* Each MD run was preceded by 500 minimization steps with the conjugate gradient method to optimize the ion and solvent molecules position, keeping the protein atom coordinates fixed. Next, 500 minimization steps were performed on the protein atoms only and finally 1000 steps were applied to the whole molecular system. The minimization was followed by a 400ps MD run at 300°K while keeping the C_α atoms fixed. Then all atoms were left free to move. The first 5 ns of the MD runs were considered as equilibration phase and were discarded from the analysis. The production phase consisted of about 45 ns for native, cleaved and latent forms, respectively, and we collected the coordinates every 1 ps. The occurrence of a partial opening in the breach region of the native protein prompted us to extend the relative MD simulation up to about 100 ns, to better study the consequent conformational rearrangement.

Supporting Table A2. Computational analytical tool.

- The CD data were analysed in terms of secondary structure fractions by the two popular programs CONTINLL, CDSSTR with the aid of the software package CDPro [Sreerama and Woody, Analytical Biochemistry 287 (2000) 252-260]. The spectra were fit by comparison with 4 different protein sets, composed of soluble and denatured proteins.

- The secondary structure elements were assigned from the average structure over 45 ns by using the STRIDE algorithm [Frishman and Argos, Proteins 23 (1995) 566-579].

- The Solvent Accessible Surface Area (SASA) was calculated by the NACCESS program [Hubbard, NACCESS. 2.1.1. Univ. Manchester (UK), 1996], which estimates the accessible area by virtually rolling a probe of 1.4 Å around the molecular Van der Waals surface. This program is an implementation of the method of Lee and Richards [Lee and Richards, J. Mol. Biol. 55 (1971) 379-400]. All the SASA calculations were performed on the protein heavy atoms. The SASA analysis was performed on conformations separated by 0.2 ns along the first 45 ns of each trajectory.

- Hydrogen bonds analysis was carried out using a VMD [Humphrey et al. J. Mol. Graph. 14 (1996) 27-38] plugin. Hydrogen bonds (HB) were defined with a cutoff distance of 3 Å between the donor (D) and the acceptor atom (A) and a D-H-A cutoff angle of 30º. The bond was classified as a salt bridge when the oxygen atom of acidic residues (Asp and Glu) and the nitrogen atoms of basic residues (Arg, Lys and the protonated His) are within the cut-off distance 3.0 Å.

- Most of trajectory analyses, such as distances, RMSD and contacts, were carried out by the ptraj module in AmberTools1.5 [Case et al. J. Comput. Chem. 26 (2005) 1668-1688].

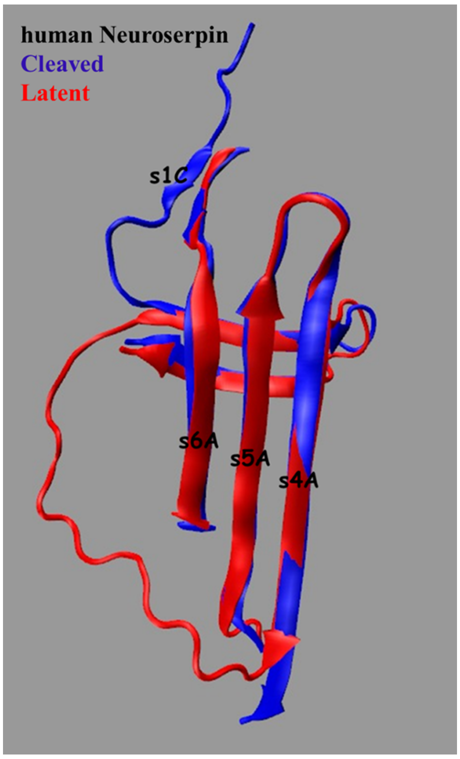

Supporting Figure A5. Modeling of latent NS.

Superposition of the relevant part of the peptide chain of the cleaved (blue) and latent (red) models. The insertion of RCL as strand s4A in the latent NS was modeled fitting the β-sheet A of the cleaved structure.

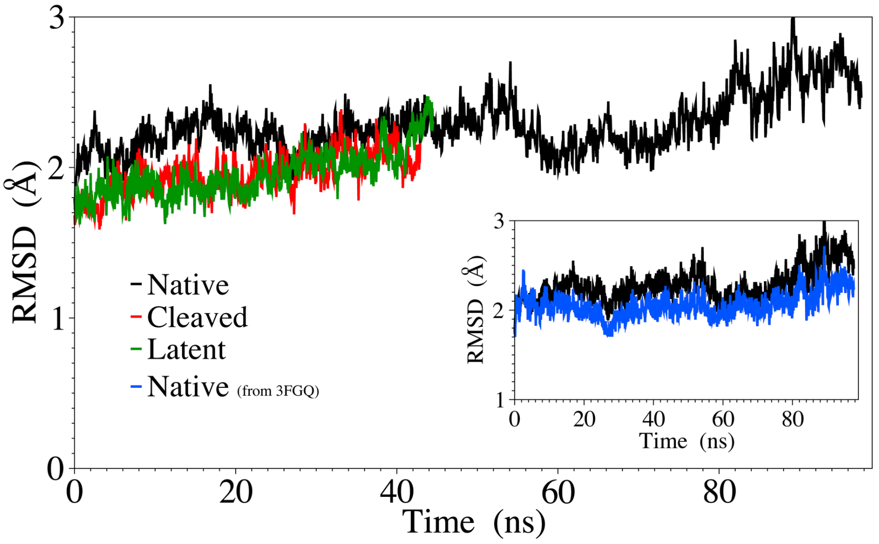

Supporting Figure A6. C_α-RMSD as a function of simulation time.

C_α-RMSDs for the native (black), cleaved (red) and latent (green) NS. The RCL, ω loop and gate residues were not included in the RMSD, as well as other unresolved x-ray domains, as they are highly mobile. The three NS conformers are relatively stable throughout the 45 ns long trajectories with RMSD values within 2.3 Å. The native NS has a larger degree of flexibility, and is also more rapid in reaching this RMSD level. In the longer run, the RMSD of native NS exhibits a further increase after 60 ns, reaching a value of about 2.7 Å. A deeper inspection of individual structural elements suggests that such an increase is mainly related to a rearrangement of strands s1A and s1B.

Inset: C_α-RMSD for the native NS with respect to the two available crystallographic structures, PDB ID 3F5N (black) [15], and PDB ID 3FGQ (blue) [28]. We note that the MD trajectory remains consistently closer to the higher resolution x-ray structure (RMSD <2.5Å, blue curve), even though the starting MD structure was taken from the lower resolution one (RMSD <2.7Å, black curve).

**Time-averaged MD features**	**Native**	**Cleaved**	**Latent**
C_α-RMSD (Å)	2.20 ± 0.1	2.00 ± 0.1	2.00 ± 0.1
Non-native contacts	115 ± 11	80 ± 10	90 ± 13
Radius of gyration (Å)	22.3 ± 0.1	21.8 ± 0.1	21.4 ± 0.1
**Radius of gyration from x-ray data (Å)**	21.88	21.41	

Supporting Table A3. Time-averaged features from MD simulations.

Time-averaged values of MD parameter are reported for native, cleaved and latent NS. The larger values for the native NS suggests a greater conformational flexibility than the cleaved and latent forms.

A sensitive way of detecting changes in the structure of a protein during a simulation consists in monitoring the native contacts between residues, defined as those found in the crystal structure of the protein. We consider two residues to be in contact if their C_α atoms are within a distance of 6 Å. The non-native contacts can arise both from disrupted native contacts as well as from completely new contacts.

The time-averaged values for the radius of gyration are perfectly comparable with the values obtained from x-ray data and do not show any trend. The radius of gyration of cleaved NS is slightly increased with respect to the structurally similar latent NS, due to two extra charged end-chains that remain fully exposed to the solvent.

Salt bridges	Native	Cleaved	Latent
Arg36 - Asp305	85*	74*	80
Arg38 - Glu268	88*	85*	86
Arg73 - Glu86	69	**94***	91
Arg73 - Glu61	—	**81***	—
Arg195 - Glu197	—	—	52
Arg259 - Glu261	52	—	—
Arg294 - Glu346	—	85*	—
Arg362 - Glu289	71	—	—
Arg362 - Glu330	—	53	—
Arg383 - Glu243	81	83*	81
Arg384 - Glu249	66	—	—
Arg384 - Glu250	—	**86***	**72**
Arg384 - Glu92	—	**75**	**92**

Supporting Table A4. Salt bridges in MD simulations.

Salt bridges in native, cleaved and latent hNS, shown as percentage occupancy during the whole simulation. Only strong salt bridges, involving arginine with two bonding sites, are reported. Complex salt bridges are highlighted in bold font. The asterisk indicates that the salt bridge also occurs in the crystallographic structure of Ricagno et al. [15]. The Arg384-Asp249 salt bridge, found in the native conformer, is analogous to the Arg384-Glu250 found in the cleaved and latent cases.

While many salt bridges in all the NS conformers with similar occupancy, as well as in the native and cleaved x-ray structures, the cleaved conformer has a higher number of them and two complex salt bridges, involving the triads Glu86-Arg73-Glu61 and Arg384-Glu92-Glu250, also present in latent NS.

In the native NS a further strong salt bridge is found between Lys206 and Glu209. The latter helps decresing the local flexibility of the gate region, which shows larger fluctuations for the cleaved and latent NS (1.7-3.6 Å) than for the native NS (1.3-1.7 Å).

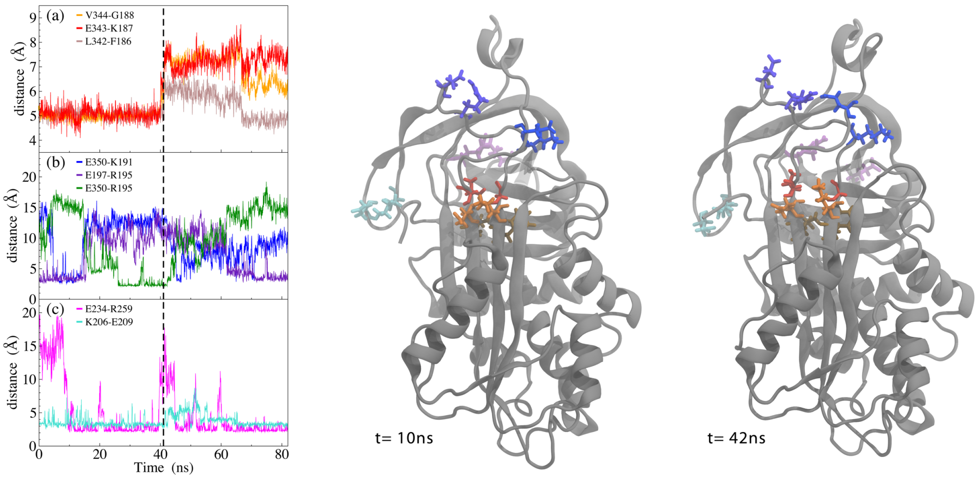

Supporting Figure A7. The broadening of the breach region. Left panel: Distances of some residue couples (as in the legend) from three important regions: (a) breach, (b) hinge, (c) gate. Central and right panels: Interaction networks at 10 ns (center) and 42 ns (right). The residue couples are highlighted in matching colours in all panels.

A partial opening of the breach region in the native NS conformer occurs around t=42 ns, while no similar events were detected in the trajectories of the cleaved and latent NS. Such observation prompted us to extend the relative MD simulation up to about 100 ns, to better sample the associated conformational rearrangements. The distances between C_α atoms of three pairs of residues (344-188, 343-187, 342-186), belonging to the s5A and s3A strands, increase abruptly at t=42 ns to reach a value of about 7 Å in the case of the two topmost pairs, and of about 6 Å for the bottom pair. The latter returns to the previous distance at t=66.7 ns, while the topmost pairs of residues remain separated (panel a).

This event is accompanied by the formation and disruption of hydrogen bonds and salt bridges both in the breach region and in other regions of the protein (panels b and c). In the hinge region e.g., residue Glu350 swaps salt-bridge mate, from Arg195 to Lys191, to remain eventually unbounded, which can be considered as a favorable circumstance for the RCL insertion (panel b). Moreover, we note a temporary breaking of the salt bridge Glu234-Arg259 between the ω loop and the gate-turn 1, and a perturbation of the temporary loosening of salt bridge Lys206-Glu209 on the other arm of the gate, the gate-turn2 (panel c). However, a more severe disruption of the bonding network is probably required to obtain a relevant loosening of the gate region.

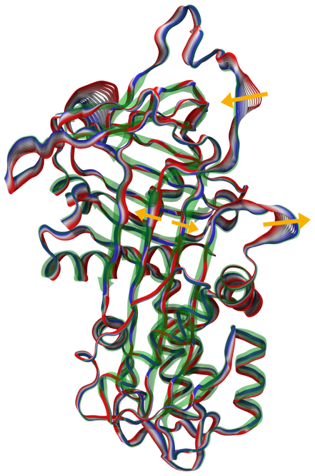

Supporting Figure A8. Essential modes in native NS. Superimposition of several time-frames, obtained by projecting the C_α motion on the third eigenvector of native NS. The yellow arrows are used as qualitative indicators of the motion direction (from red to blue).

The most important collective motions of the protein have been identified by the essential modes [Berendsen and Hayward, Curr. Opin. Struct. Biol. 10 (2000) 165-169] technique. The covariance matrix of the C_α atoms cartesian displacements has been diagonalized and the eigenvectors with the 5 largest eigenvalues have been analysed. Since, the technique suffers from the poor sampling of very slow modes of the protein, typical of molecular simulations [Balsera et al. J. Phys. Chem. 100 (1996) 2567-2572], the convergence of the sampled space was verified by computing the cosine content of the principal components, that is a marker of its diffusive character [Hess, Phys Rev E 65, (2002) 031910], by using a tool from the GROMACS suite [Hess et al. J. Chem. Theory Comput. 4 (2008) 435-447]. In fact, in the case of a poor sampling of the conformational, the random (normal) diffusion produces cosine-shaped principal components (PCs) [Hess, Phys Rev E 65, (2002) 031910], which can mistakenly be interpreted as a transition of the system from one state to another. The Wordom package [Seeber et al. Bioinformatics 23 (2007) 2625-2627] was used to build and diagonalize the covariance matrix of the coordinate fluctuations. The visualization of the motions along each eigenvector was accomplished with the VMD molecular package.

By looking at the first five principal components (PC1 … PC5), we found values of cosine content greater than 0.5 for PC1 and PC2, and values less than 0.5 for PC3-PC5, for all three forms of NS (Table A2). We focused on the native conformer, whose trajectory features interesting dynamical events, and we considered in particular the third principal component of the Cα motion (Fig. 8). The dominant motions of native NS in PC3 are a partial opening of the parallel strands of the β-sheet A in the breach region and a pincer motion, involving two large correlated regions of the protein. The first region comprises the RCL and the loop Ω, the helices G-H, the turn linking 4B to helix G, and the gate, whereas the second region comprises the strands s1A, s2A, s3A, the helices B-C-D-E-F, the turn linking s2A to helix D, and the two loops in the gate region. However, such a suggestive correlation between the opening of the breach region and the large movement of the loops region should not be given a causal relationship, since loop regions, not hindered by strong intramolecular interactions, are allowed to perform large coherent movement.
